# Blockchain technology and its impact on sustainable supply chain management in SMEs

**DOI:** 10.7717/peerj-cs.2466

**Published:** 2025-03-04

**Authors:** Chao Fang, Nazir Ullah, M. Batumalay, Waleed Mugahed Al-Rahmi, Fahad Alblehai

**Affiliations:** 1 School of Economics and Management, Bengbu University, Bengbu, Anhui, China; 2 School of Management Science and Engineering, Nanjing University, Nanjing, China; 3 Faculty of Data Science & IT, INTI International University, Nilai, Negeri Sembilan, Malaysia; 4 Department of Management Information System, College of Business Administration, Dar Al Uloom University, Riyadh, Al Falah, Saudi Arabia; 5 Computer Science Department, Community College, King Saud University, Riyadh, Saudi Arabia

**Keywords:** Blockchain, SMEs, SDGs, Supply chain, COVID-19, Process innovation

## Abstract

The COVID-19 pandemic has had a significant impact on small and medium-sized enterprises (SMEs), leading to disruptions in supply chains, financial losses, and closures. To overcome these challenges, organizations, including those in developing economies like Malaysia, are turning to blockchain technology as a solution to enhance traditional supply chain management frameworks. This study aims to identify the factors that influence the acceptance of blockchain technology among SMEs. By drawing on established adoption theories such as the technology acceptance model (TAM), diffusion of innovation (DOI) theory, and theory of planned behavior (TPB), the researchers developed a research framework. They utilized partial least square structural equation modeling (PLS-SEM) to analyze the causal relationships between different constructs and test their hypotheses. The findings confirmed that the constructs of the technology acceptance model, specifically perceived usefulness, perceived ease of use and attitude were significantly associated with the intention to use blockchain technology. Additionally, the constructs of the diffusion of innovation theory, relative advantage and compatibility, showed significant associations with perceived ease of use, while complexity had a negligible relationship with perceived usefulness and perceived ease of use. The construct of subjective norms from the theory of planned behavior exhibited a significant relationship with perceived usefulness and an insignificant relationship with intention to use. Finally, perceived behavioral control demonstrated a positive relationship with intention to use. The study’s findings provide valuable insights for blockchain developers and organizations aiming to make informed decisions regarding the application of blockchain technology as a process innovation in SMEs.

## Introduction

The current business model has been disrupted by the COVID-19 outbreak, making actors in the food supply chain more vulnerable. COVID-19 has affected the worldwide economic process, and raised concerns among many about the ability of food firms to survive, which may ultimately have an impact on food safety, particularly in relation to availability, access, and utilization (Iranmanesh et al., 2023). The current innovations in virtual industrial technology and the manifestations of diversified gadgets in Industry 4.0 have enabled firms to create, utilize and exchange data, enabling quicker, more flexible, and more efficient processes. In the post-pandemic era, these enhanced processes have developed digital market ecosystems of interest by implementing information and communication technologies. Such processes provide innovative ways to bolster organizations in various industries to strengthen technological resources and support customer needs effectively, contrary to conventional corporate environments. In the modern system, every item is connected, developing a digital imperative for firms to create transformative technology, enabling the use of these improvements. A study by Bär, Herbert-Hansen & Khalid (2018) indicates that there is only a little work emphasizing how an organization could evaluate the future benefits of Industry 4.0 innovations and their effect on service and manufacturing operations. In the post-pandemic era, disruptive technologies such as blockchain play a crucial role in processing the exchange of information for sustainable supply chain management. Conventional supply chains are geographically distributed, and link maintenance is required; the coordination activities between supplier and customer are no longer self-sufficient. There is also a research gap in strategy implementation. Identifying this gap, the current literature has emphasized the significance of emerging technology in manufacturing and service operations, such as big data (Govindan et al., 2018), artificial intelligence (Adel, Elhakeem & Marzouk, 2022), and blockchain technology. In the post-pandemic era, firms investing in emerging technologies have recognized their ability to become competitive and reduce production costs. They have to move from running unorganized storage to integrating operations and maintenance development around the internal end-to-end procedures and connections of potential clients. In addition, implementing innovations and enabling technology would have to be blended adequately to achieve a comprehensive and triggering effect.

Emerging technologies have created modern corporate communication networks, such as digital company ecosystems. For example, in 2016 Walmart pursued blockchain for item tracking through improved supply chain transparency. Using the pilot project to track mangoes from an orchard in Mexico to the United States usually takes 6 days, 18 hours and 26 minutes. However, using a blockchain-based network took just 2.2 s (Kamath, 2018). Similarly, some related projects, such as tuna tracks created by the Worldwide Fund for Nature, are blockchain prototypes for mapping sorted contract documents through the entire supply chain. Small and medium enterprises (SMEs) can no longer depend on traditional processes in a digitally transformed world. During the post-pandemic era, SMEs play an important role in Malaysia’s diverse market environment (Jaafar, 2018); SMEs economy to grow by 4.5% in 2021 and 5.8% in 2022. Blockchain is one significant technical advantage in the sustainable supply chain management realm for SMEs. Manufacturing and service operations enabled for actual data collection have end-to-end visibility that permits management to use a high volume of data and make informed decisions. In general, decentralized ledgers could be the ultimate answer to improving the SMEs supply chain’s existing problems by resolving traceability, tracking, and supporting SMEs in the digital transformation.

**Figure 1:**
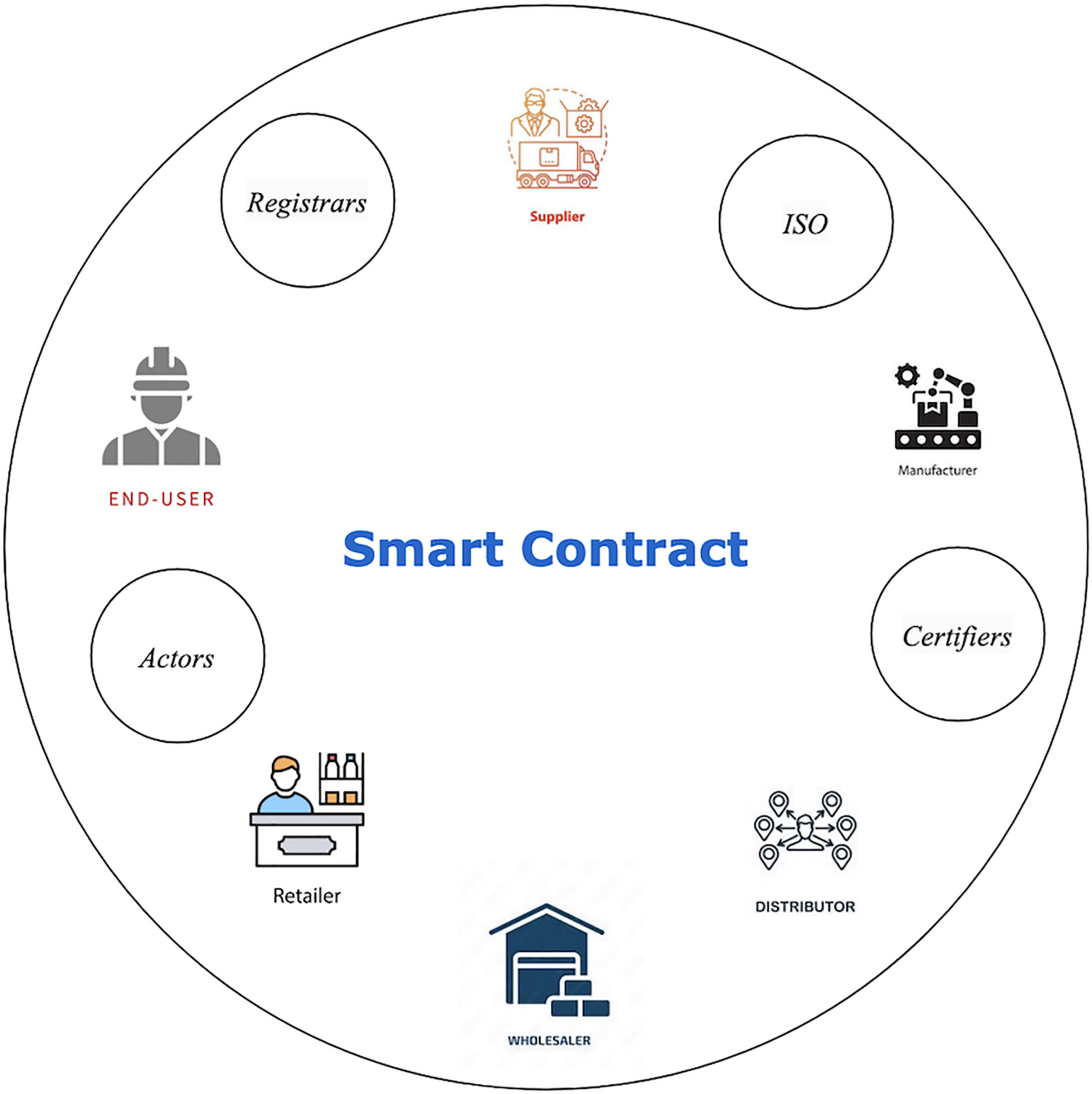
Supply chain transformation. Created in diagrams.net.

The use of blockchain guarantees data privacy, authenticity, and smart contractual dealings for a trustless business environment, and plays a substantial role in the sustainable supply chain management of SMEs. A typical illustrative of one traditional supply chain conversion to a blockchain-based supply chain is shown in Fig. 1. In the blockchain-based sustainable supply chain management (SCM) system, four main actors play roles that are not seen in traditional SCM: *Registrars*, which offer unique identities to participants in the system network; *Standard Organizations* that identify standards such as Fairtrade for blockchain-based supply chain networks and technical specifications; *Certifiers*, who certify actors for inclusion in the network; and *Actors*, including manufacturers, retailers and end-users, who must be registered by a listed certifier to hold the trust of the blockchain-based supply chain network. At least five main commodity dimensions can be highlighted and detailed by the blockchain-based network:

- Nature (what type of a product it is).
- Quality (how it is).
- Quantity (how much).
- Position (where).
- Ownership of each product

This way, all the information is stored in the distributed ledgers through a smart contract with verifiable updates. The information lets consumers examine the uninterrupted custody chain and transactions, from raw materials to the final sales. For SMEs, the most interesting feature of the blockchain is that each actor in the network has to seek permission to transfer the product’s ownership through smart contract agreements and consensus algorithms. Therefore, blockchain has a pivotal role in supply chain operations and the management of goods and financial transactions between various network parties.

Due to inadequate transformational processes, the technological advantages of digitized sustainable supply chain management for SMEs are still largely undiscovered. However, one innovative technological advantage underneath SMEs has blockchain technology. According to a study by Queiroz, Telles & Bonilla (2019), there is still a research gap in the theoretical integration background for blockchain supply chain applications. Still, the disruptive effects are evident, despite the innovative technology’s initial phase. The existing research on blockchain adoption in SMEs is mostly focused on developed economies like the US, UK, and EU. However, this study aims to fill the gap by focusing on blockchain acceptance in SMEs in developing countries. While past research on blockchain technology has primarily focused on the technology-organization-environment context and literature review form, our empirical study proposes a model that integrates the technology acceptance model with diffusion of innovation theory and theory of planned behavior. Through this inquiry, we aim to shed light on whether critical factors can influence the acceptance of blockchain among SMEs in Malaysia. Many academic researchers have addressed how blockchain applications can achieve supply chain goals on a conceptual level, but in the post-pandemic era, few have focused directly on SMEs. Hence, to the greatest of our knowledge, it’s one of the primary studies that assessed blockchain as a scientific advance by using a novel methodology for Malaysia that can enhance the various priorities for SMEs. In the post-COVID-19 era, blockchain adoption will greatly help cost efficiency, improve inventory replacement, strengthen marketing strategy, and increase product safety. Blockchain applications in SMEs will ultimately aid the retailers in tracking the stock in real-time and lessen labor costs. Furthermore, in the production domain, it will aid in implementing the anticounter measures.

To validate our model and test hypotheses, we employ the partial least square structural equation modeling technique. This technique is well suited for real world research initiatives as it does not rely on any presumptions about data distribution. It allows us to estimate composite models with multiple factors, indicator constructs, and structural routes without making distributional assumptions about the data. This approach is favored by academics for its flexibility and ability to handle complex models. Moreover, our study focuses on providing insights into best practices for effectively utilizing blockchain technology in SMEs. By integrating the technology acceptance model with the diffusion of innovation and theory of planned behavior, we aim to identify the drivers of improvement and guide SMEs in their adoption journey. The insights gained from early adopters can help SMEs in persisting with the proof of concept for the digitization of production and distribution processes. It is worth noting that blockchain technology has advanced to the concept testing stage, and is being deployed in various industries. In SMEs, for example, blockchain technology has the potential to enhance operational efficiency through its traceability capabilities. It can improve safety, lower prices for consumers and businesses, and foster trust. Furthermore, a trust model built on blockchain technology can facilitate digital rights management for supply chain management. By integrating the technology adoption theories, this research aims to contribute to the understanding of blockchain adoption in SMEs and provide valuable insights for their successful implementation.

Therefore, the main drive of the current study is to respond to the question of whether the assimilation of traditional adoption theories could affect the implementation of blockchain in manufacturing and service operations in Malaysia. It will also investigate how SMEs could manage and advance digital scenarios to run on cycles of digital transformation for operations management. The findings are expected to aid experts in evaluating operational and technical issues affecting SMEs amid globalization and market integration. Therefore, the core driver of this research study is to answer the following queries:

RQ1. What factors drive the Malaysian SMEs’ intention to implement blockchain in the manufacturing and service operations?

RQ2. Among the factors, which are more closely associated with adoption intention?

The structure of the study is ordered as follows. ‘Literature Review’ explains the theoretical background of a study. ‘Development of Proposed Model’ depicts hypothesis development. ‘Methodology’ explains the research methodology and clarifies the study findings. ‘Discussion’ provides a summary.

## Literature review

### Blockchain technology

Several tactics have been implemented to create trust and decrease knowledge asymmetries between manufacturers and consumers. The most prominent among manufacturers who pledge to adhere to certain predetermined provisions is expanding a common label. A common label has been used to assure customers of the particular values of the products they have purchased. The common label has a range of flaws and severe limitations. First, opportunistic methods are listed. Some manufacturers claim to follow the instructions of the common labels, but in fact, they do not follow them. Furthermore, rivals often employ fake tactics, whether by using deceptive labels or complex alternatives, such as tags and pictures from third countries to confuse or mislead customers. Many of these barriers have been overcome by the evolution of digital technologies (Dong et al., 2023). Blockchain technology is aimed at reinforcing trust and creating new dealings between manufacturers and consumers. In the post-pandemic era, blockchain and the Internet of Thing (IoT) are key components of this newest wave. The ability to offer digital identities to every product and component is at the heart of this modern digital revolution. As a result, markets will become more transparent in the post COVID-19 era. The Internet of Things would build marketplaces by finding and balancing supply and demand in real-time. These dynamic digital markets will rapidly extend this transition’s scope by building on the groundwork laid by gadgets and social networks. They will generate peer-to-peer economic models and encourage shared markets.

Blockchain was first used in the application of Bitcoin, the primary decentralized digital currency that removes the middle person from the transaction. Blockchain is also used in many domains due to its benefits in distributed ledger data management, and the possibility of inspection trials. Engineering management, decentralized governance, and health care management are among the sectors that have adopted it since 2014. Blockchain provides peer-to-peer data sharing, immutability, and computational logic. However, the precise method depends on consensus algorithms. There are three phases of blockchain applications. Blockchain 1.0 deals with cryptocurrencies, such as Bitcoin; Blockchain 2.0 incorporates smart contracts for selling processing; and Blockchain 3.0 provides an excessive level of control through an autonomous network based on smart contracts with certain guidelines. Everyone could join the network in permissionless blockchain, such as Ethereum and Bitcoin. It is primarily built on a consensus algorithm known as proof of work, in which every user can certify blocks. While in permissioned networks, such as Hyperledger Fabric, only members can validate the transactions. Every node generates blocks in the proof of authority and proof of stake algorithms. In addition, based on predefined guidelines, the consortium is a mixture of permissioned and permissionless blockchains. One latent use of blockchain is to eliminate the trust-related problems associated with technology, hence cutting costs in the transaction processes. Due to the dynamic structure of supply chains in the digital period, blockchain is intended to increase speed. It would be the primary data source, and connect all supply chain processes. Blockchain applications help bring transparency and increase traceability in the traditional supply chain system. Subsequently, in the post-pandemic era, blockchain adoption could increase cost efficiency, high product security, data sharing, teamwork planning, and operation integration (Dutta et al., 2020).

### Blockchain based supply chain system

Malik et al. (2019) conducted research on supply chain challenges in terms of traceability and trust. They proposed a blockchain-based sustainable multi-layer trust management system to inspect exchanges between supply chain users, and vigorously assign trust scores based on these dealings. Subsequently, it lessens overhead regarding latency and throughput when related to a simple distributed ledger technology (DLT)-based supply chain model. Another study by Dobrovnik et al. (2018) explains potential applications for adoption, and provides a framework for classifying blockchain openings in the logistic industries. Their study aids managers in systematically evaluating where to start building organizational capabilities to deploy blockchain effectively. The study of Sahebi, Masoomi & Ghorbani (2020) thoroughly examines the barriers to blockchain technology (BCT) acceptance in the context of humanitarian sustainable supply chain management. The barriers were studied using an integrated methodology that included Fuzzy Delphi and the Best Worst Technique. Regulatory ambiguity, a lack of expertise and high sustainability costs were identified as major barriers. Their findings aid policymakers with important advice to improve their solutions. Kim & Laskowski (2018) discussed blockchain and its potential applications in the ontological engineering of supply chain provenance. Their study focused on potential effects to demonstrate techniques grounded on formal and informal ontology. Their research analyzed a traceability ontology and translated some of its representations into Ethereum-based smart contracts that performed a provenance trace and imposed traceability constraints. Another study by Omran et al. (2017) proposed a blockchain-based sustainable supply chain finance model, with automatic reconciliation and quality identified as the supply chain’s primary value drivers. Blockchain was created with these goals in mind. Their findings contributed to future advancements of suitable supply chain finance solutions based on blockchain revolutions. The study by Tian (2017) developed a hazard analysis blockchain-based sustainable traceability system for food tracing. They also unveiled BigchainDB, a novel model aimed at filling the gap in the distributed ledger system at scale. Their research finishes with a discussion of a use case and the challenges of implementing blockchain in future food supply chain traceability systems. The study by Nakasumi (2017) developed a blockchain-based approach to address sustainable supply chain difficulties, such as information asymmetry. Their research contributes to asymmetric information between the organization, which disrupts planning algorithms. The study of Korpela, Hallikas & Dahlberg (2017) presented a cloud-based solution to solve the gap between commerce readiness and current functionalities. Smart contracts are the most critical for revolutionizing the digital supply chain through blockchain integration, according to their results. The study of Toyoda et al. (2017) presented a blockchain-based product ownership management system for anti-counterfeit goods using radio-frequency identification (RFID). Their findings revealed that normally the expense of handling the ownership of a commodity with up to six transferals is less than US $1. The study by Abeyratne & Monfared (2016) offers a thorough analysis of the current status of blockchain, emphasizing its potential to significantly transform the manufacturing supply chain of the future. The authors investigate various applications of BCT, showcasing its considerable benefits for improving supply chain operations in manufacturing. They outline a progressive vision for manufacturing systems that leverage BCT, using the production of cardboard boxes as a concrete example to illustrate how this technology can be integrated into a global supply chain network. Furthermore, the article addresses the critical requirements and challenges that need to be tackled to successfully implement BCT in future manufacturing initiatives.

### Technology adoption models

In the field of industry, technical advancements often play a critical role. Technological advancement also promotes information sharing. But technology is of no use before and until it is approved or used. Although the word adoption is used discretely, the masses should think of diffusion as adoption. The adoption of technology will enable diffusion. So, identifying the adoption of technology is of paramount importance. The study of Carr (1999) described the adoption of technology as the stage of selecting technology that a person or group can use. Adopting technology may also be characterized as the ability within a community of customers to use technology to their advantage. Recent findings have shown that technology implementation is not linked to technical features alone, but has developed as an even more difficult procedure, including users’ attitude and personality dimensions, and making several conditions simpler. Previous research has shown that technology adoption is not solely related to the characteristics of the technology itself, but is a complex process involving customer characteristics, trust, and various facilitating environments. To understand the dynamics influencing the decision to implement blockchain technology in SMEs, several conceptual models based on adoption theories have been developed. These models, such as the technology acceptance model and the theory of planned behavior, have gained popularity in the literature due to their success in determining IT implementation. However, it is important to note that these theories have mainly been developed and tested in Western countries, with limited research conducted in developing nations like Malaysia. Therefore, it is crucial to study the adoption of blockchain technology specifically in the context of SMEs in Malaysia. This research would shed light on the factors influencing adoption, and provide valuable insights for organizations considering the implementation of blockchain technology. The recent literature on blockchain technology by using technology adoption models is presented in Table 1.

**Table 1:** Adoption models.

Author	Model used	Major findings
Clohessy, Acton & Rogers (2019)	Technology organizations environment	The study findings confirm that organizational readiness, top management, and organizational support are considered as the key interests when implementing disruptive technology by organizations.
Queiroz & Wamba (2019)	Technology acceptance model, Social support theory, Perceived trust, Perceived risk	From the study results, it is revealed that social media raises perceived trust and the ability to use digital currencies.
Moore & Benbasat (1991)	Technology acceptance model/theory of planned behavior/technology readiness index	The study findings indicate that integrating the technology acceptance model (TAM) with theory of planned behavior showed a substantial influence on blockchain adoption. Conversely, the technology readiness index had no considerable relationship during blockchain adoption in the supply chain.
Davis (1989)	Diffusion of innovation theory	According to the findings of the study, enterprises would grow as the first to adopt blockchain; for instance, oil trading will supply numerous provider layers. The study expected that a blockchain endorsement by one organization will place standardizing pressure on multiple supply network members, using the rancher as an example.
Fishbein et al. (1980)	The unified theory of acceptance and use of technology	The study concludes with supply chain consequences of disruptive technologies as elicited by prior literature and theory for supply chain traceability.
Wamba, Queiroz & Trinchera (2020)	Technology acceptance model/diffusion of innovation theory	The outcomes of the study aid in the understanding of blockchain applications in Fintech operations.
This study	Technology acceptance model/diffusion of innovation theory/theory of planned behavior	Our Findings adds to the limited empirical research on understanding the blockchain adoption in the SMEs for post-COVID-19. Based on Study findings, it’s indicated that technology acceptance model constructs matters most during blockchain implementation in the SMEs-Malaysian context.

## Development of proposed model

### Proposed model

**Figure 2:**
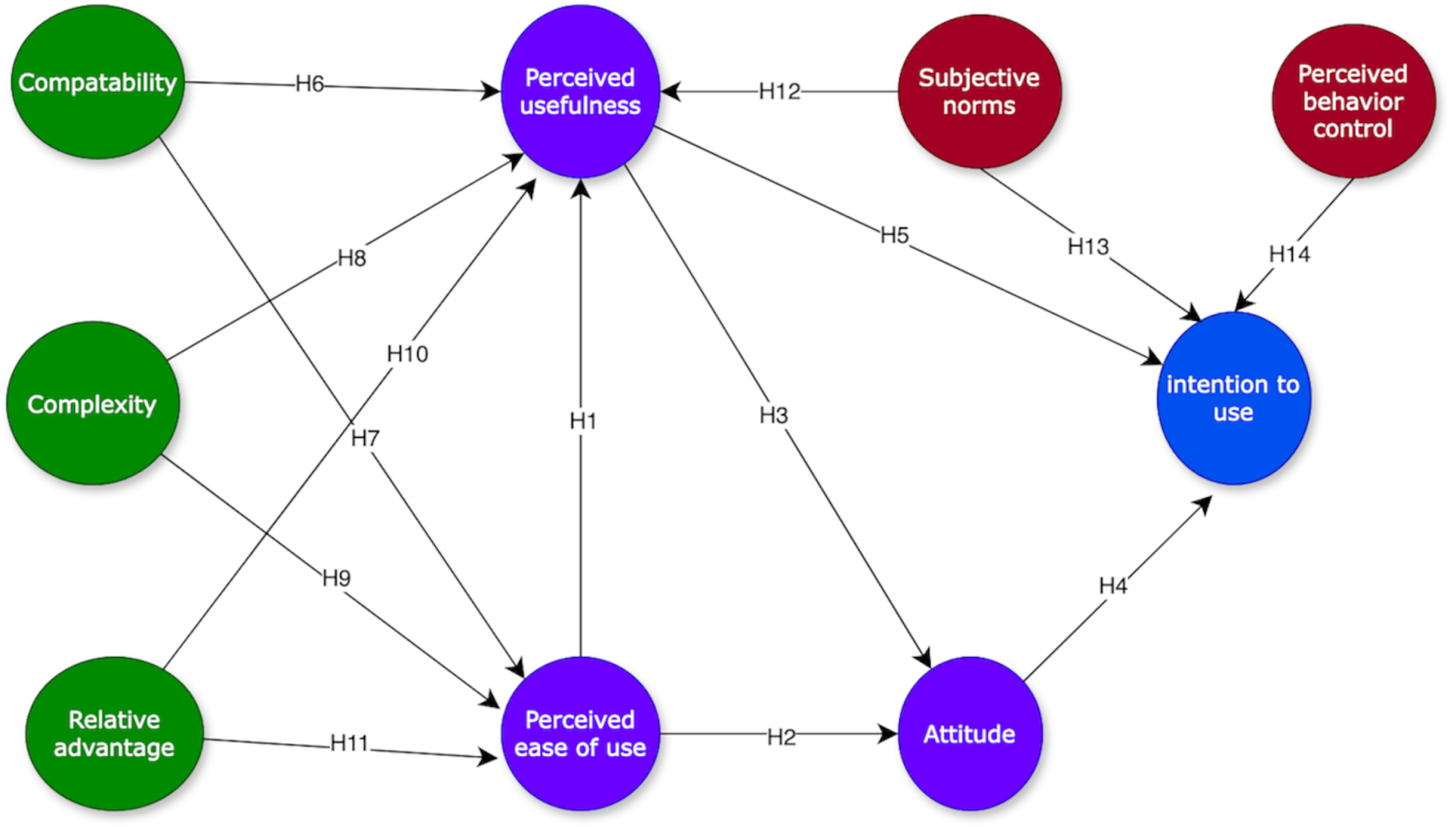
The proposed model.

The use of blockchain in the supply chain has been researched and written by some researchers. Many of the basic approaches that were deployed involved the technology acceptance model (Kamble, Gunasekaran & Arha, 2019), Technology-Organization-Environment framework (Clohessy, Acton & Rogers, 2019), and the unified theory of acceptance and use of technology (Queiroz & Wamba, 2019). To supplement these prior studies, the present study shows how the technology acceptance model can be integrated with the diffusion of innovation theory and theory of planned behavior for the sustainable supply chain management of SMEs. Accordingly, the adoption of blockchain in development is still nascent, and its market potential has yet to be grasped entirely. Ideally, the diffusion of an invention assessment does not have any apparent connection with technology acceptance model, but mutually share some primary factors. It was uncovered that the relative advantage in the diffusion of innovation theory is the same as the idea of the perceived usefulness in technology acceptance model. The complexity paradigm in the diffusion of innovation theory captures the perceived ease of use in the technology acceptance model, while the sign is reversed (Moore & Benbasat, 1991). In addition, in terms of complexity, technology acceptance model and diffusion of innovation theory indicate that individual purpose is partially dictated by how challenging it is to grasp or practice the technology. Moreover, the less difficult it is to use, the more likely it is for a person to consider it. Compatibility is associated with prior experience with a technology’s performance, whereas the desire to seek observation is related to the availability of appropriate opportunities for experience. These factors relate to previous experience with technology, or opportunities to experience the technology being considered. Compatibility and the ability to observe can be regarded as external variable quantity that directly affect the factors in the technology acceptance model. Finally, constructs from the theory of planned behavior, such as subjective norms and perceived behavior control, which incorporate user experience dimensions, when linked to technology acceptance model constructs, offer further insights into technology acceptance. The basic conceptual model is exhibited in Fig. 2.

### Hypotheses development

#### Technology acceptance model

The technology acceptance model (TAM) is proposed by Davis (1989). TAM has been verified empirically as a method for estimating the use of technology, and has emerged in the literature as the dominant model. One of its fundamental strengths is technology acceptance model predictive capacity. The technology acceptance model depends on two clear factors, perceived ease of use and perceived usefulness, irrespective of how much the end consumer is compelled to recognize and use the information system. Perceived ease of use is the extent to which a specific information system claims to be safe from emotional constraints, and perceived usefulness is the degree with which a person identifies the usage of different structures to boost one’s performance. In addition, attitude is the positive or negative sentiments of an individual about a target behavior. Subsequently, attitude is suggested as a multidimensional factor, often measured in overall affective evaluation (Fishbein et al., 1980). The results from the previous studies confirm that perceived ease of use is positively associated with perceived usefulness (Wamba, Queiroz & Trinchera, 2020; Karamchandani, Srivastava & Srivastava, 2019; Folkinshteyn & Lennon, 2016). Perceived ease of use is positively associated with attitude (Al-Rahmi et al., 2020, 2019). Perceived usefulness is positively associated with attitude (Kanchanatanee, Suwanno & Jarernvongrayab, 2014). Attitude is significantly associated with intention to use (Alalwan et al., 2019; Alamri, Almaiah & Al-Rahmi, 2020; Alenazy, Al-Rahmi & Khan, 2019). Subsequently, perceived usefulness is significantly associated with intention to use (Gefen, Karahanna & Straub, 2003; Ullah et al., 2020). Correspondingly, the present study expects that technology acceptance model constructs will substantially associate with intention to use blockchain in the supply chain management of SMEs. Therefore, we postulate the following hypothesis.

H1. Perceived ease of use is positively associated with perceived usefulness of blockchain.

H2. Perceived ease of use is positively associated with attitude towards using blockchain.

H3. Perceived usefulness is positively associated with attitude towards using blockchain.

H4. Attitude is positively associated with intention to use blockchain.

H5. Perceived usefulness is positively associated with intention to use blockchain.

#### Diffusion of innovation theory

The diffusion of innovation theory is proposed by Rogers (1995). Innovation diffusion theory has been extensively applied in advertising, social media, farming, and system engineering. An innovation is awareness, practice or item that a person perceives as new for adoption and use. Moreover, diffusion is the mechanism by which the social network members transfer an idea through numerous systems over time. Accordingly, the distribution of innovation theory claims that potential consumers decide to accept or decline grounded on prejudices they developed about the innovation. In the current study, we select the three essential features of diffusion of innovation theory: compatibility, complexity, and relative advantage. Compatibility is the extent through which innovation is regarded as compatible with the potential end consumer existing viewpoints, previous prospects and obligations. In cryptocurrency adoption, compatibility is measured as one of the prominent constructs. Complexity is the apparent degree of difficulty on the part of end-users in recognizing technologies. Complexity depends on the difficulty of the application of technology and the technology itself. Usually, a high level of complexity confuses individuals and makes it difficult for them to understand new technologies, negatively affecting their adoption behavior. Blockchain adoption may partly be postponed due to its immaturity and security challenges. The following relative advantage is the extent to which an innovation is measured as better than the idea it has replaced. The current study also reflects that relative advantage is an important construct in accepting blockchain technology. Because of better transparency, cost efficiency, improving stock replenishment, and increased protection for supply chain traceability, it would offer many advantages when successfully implemented in SMEs post-COVID-19. The previous study results ratify that compatibility is substantially associated with perceived usefulness (Kumpajaya & Dhewanto, 2015). Compatibility is positively associated with perceived ease of use (Chang & Tung, 2008; Wu & Wang, 2005). Complexity is negatively associated with perceived usefulness (Tandon, Kiran & Sah, 2016; Kim, Mannino & Nieschwietz, 2009; Amoako-Gyampah, 2007). Complexity is negatively associated with perceived ease of use (Tsai, Lee & Wu, 2010; Yoo et al., 2019). Finally, the relative advantage is positively associated with perceived effectiveness (Izuagbe, Hamzat & Joseph, 2016; Wang, Meister & Wang, 2008). Relative advantage is positively associated with perceived ease of use (Ong, Poong & Ng, 2008; Rose & Straub, 1998). Consequently, this study expects that diffusion of innovation theory constructs show a key position in accepting blockchain technology for SMEs. Therefore, we postulate the following hypothesis.

H6. Compatibility is positively associated with perceived usefulness of blockchain.

H7. Compatibility is positively associated with perceived ease of use of blockchain.

H8. Complexity is negatively associated with perceived usefulness of blockchain.

H9. Complexity is negatively associated with perceived ease of use of blockchain.

H10. Relative advantage is positively associated with perceived usefulness of blockchain.

H11. Relative advantage is positively associated with perceived ease of use of blockchain.

#### Theory of planned behavior

The theory of planned behavior is proposed by Ajzen (1985). It is an expansion of the theory of reasoned action. The theory of reasoned action consists of numerous different attitude concepts, such as teaching strategies, consistency approaches, and expectancy-value ideas. The technology readiness action indicates that individuals are expected to accept new technologies if they are optimistic about a subject and its peers. The theory of planned behavior discusses circumstances where people are not entirely in charge of their behavior. In the present study, we select the two main factors of the theory of planned behavior, namely subjective norms and perceived behavior control, to identify individuals’ attitudes toward blockchain adoption of SMEs post-COVID-19. Subjective norms are the impression by the individual that too many people close to him believe that the action in question should or should not be carried out. Perceived behavior control focuses on the individual’s expectations of their volume to execute a particular action to the level that it is a particular indicator which can be used to forecast actions along with perceived behavior intent. The prior study findings confirm that subjective norms are positively associated with perceived usefulness (Dalila et al., 2020). Moreover, perceived behavior control is also positively associated with intention to use the information system (George, 2004; Prati, Mazzoni & Zani, 2014). This study also suggests that the theory of planned behavior constructs considerably affect the intention to use blockchain in sustainable supply chain management of SMEs. Therefore, we formulate the subsequent hypothesis.

H12. Subjective norms is positively associated with perceived usefulness of blockchain.

H13. Subjective norms is positively associated with intention to use blockchain.

H14. Perceived behavior control is positively associated with intention to use blockchain.

## Methodology

**Figure 3:**
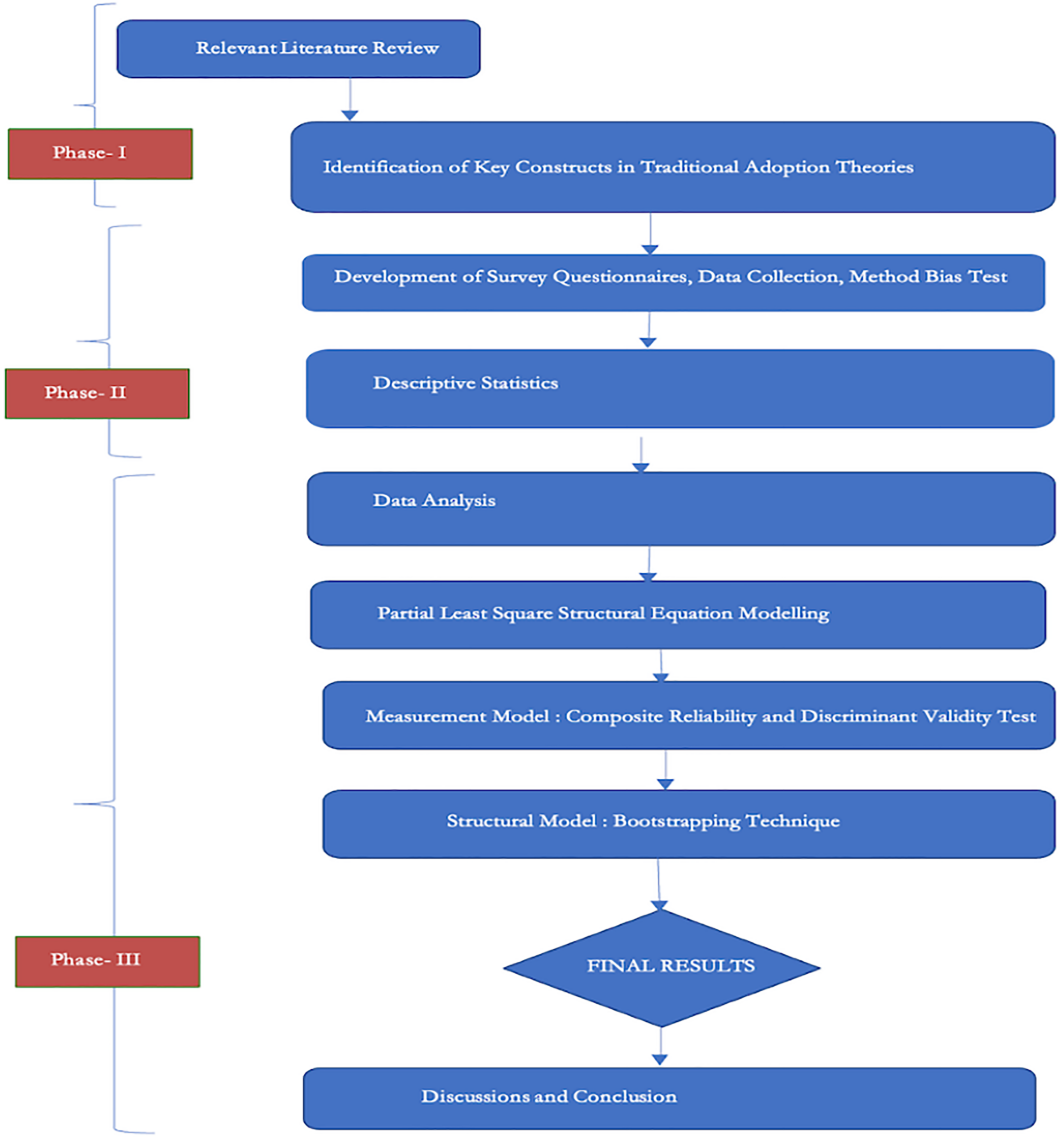
Flowchart of methodology.

This article proposed a model by integrating three traditional adoption theories and expanding their features for BCT acceptance by SMEs post-COVID-19. Partial least square structural equation modeling (PLS-SEM) was used to assess the relationship among the factors and test the hypothesis. To achieve better results for a unified proposed model, it is a technique for aiding linear and additive models commonly employed in business management studies. It has become increasingly popular in many studies due to its appropriate and reliable methodologies for examining composite models in exploratory studies. Due to their inadequate capability for complex modeling, the first generation techniques were opted out. Because of the complicated nature of structural models, such as several components with multiple indicators, PLS-SEM is widely utilized instead of covariance-based research techniques in the second generation. The analysis of Hwang et al. (2010) encourages using PLS-SEM for emerging technologies and information systems. SmartPLS is a widely used and approved method for investigating technology adoption models. The flowchart depicting the research methodology is shown in Fig. 3.

### Instrument development

A survey instrument was developed for PLS-SEM to test the correlation between constructs in the proposed model. The pilot 5-point Likert scale close-ended questionnaire was selected for the study (Croasmun & Ostrom, 2011). The five experts first checked the questionnaire’s content for clarification within Malaysian context. For the pretest, questionnaires were sent to 12 experts working in finance, logistics, and IT departments of SMEs in Malaysia. Their feedback has helped us to validate the instrument. The details of the construct measurements is presented in Table 2.

**Table 2:** Constructs measurement.

Construct	Code	Question	Adapted from
Perceived ease of use	PEOU1	In the post-pandemic, the blockchain database will be easy to use	Al-Rahmi et al. (2020), Kumpajaya & Dhewanto (2015), Alenazy, Al-Rahmi & Khan (2019)
	PEOU2	You believe blockchain technology is understandable	
	PEOU3	It will be easy to do several task by blockchain quickly	
	PEOU4	In the post-pandemic, the blockchain is easy to use than the traditional supply chain system	
Perceived usefulness	PU1	The blockchain can aid SMEs in Malaysia for persistent transactions in post-pandemic	Rogers (1995), Gefen, Karahanna & Straub (2003), Fornell & Larcker (1981)
	PU2	It can bring transparency to SMEs in post-pandemic	
	PU3	It can aid supply chain productivity in post-pandemic	
	PU4	It can improve supply chain performance in post-pandemic	
Attitude	ATT1	In your view, blockchain is essential to use for SMEs in post-pandemic	Chang & Tung (2008), Alenazy, Al-Rahmi & Khan (2019)
	ATT2	You think it will be better for the Supply chain of SMEs in Malaysia to use blockchain technology in post-pandemic	
	ATT3	You think, using blockchain is a good idea for SMEs in post-pandemic	
	ATT4	Overall, your attitude about blockchain implementation in SMEs is positive	
Compatibility	CPT1	Blockchain is compatible with manufacturing traceability and transparency in post-pandemic	Lisha et al. (2017), Amoako-Gyampah (2007), Yoo et al. (2019)
	CPT2	Blockchain is compatible with speedy transactions in SMEs	
	CPT3	Blockchain is compatible for better inventory replenishment in post-pandemic	
	CPT4	Blockchain is compatible with most features of SMEs in post-pandemic	
Complexity	CPX1	Blockchain has some technical issues like immaturity (slow and cumbersome)	Ong, Poong & Ng (2008), Rose & Straub (1998), George (2004)
	CPX2	Blockchain lacks scalability	
	CPX3	Blockchain lacks interoperability	
Relative advantages	RAD1	Blockchain will reduce the cost of SMEs in post-pandemic	Prati, Mazzoni & Zani (2014), Yoon (2016), Hwang et al. (2010)
	RAD2	Blockchain will enhance the security of SMEs in post-pandemic	
	RAD3	Blockchain will increase asset visibility in SMEs in post-pandemic	
	RAD4	Blockchain will reduce stock loss in SMEs	
Subjective norms	SN1	You believe many international firms have already achieved benefits from the blockchain technology	Gao & Bai (2014), Moore & Benbasat (1991), Chang, Witteloostuijn & Eden (2010)
	SN2	You prefer blockchain is favorable for SMEs in post-pandemic	
	SN3	You think SMEs can get a competitive edge by using blockchain technology	
	SN4	You expect that soon SMEs will use the blockchain applications	
Perceived behavior control	PBC1	Your company can buy blockchain applications	Hair et al. (2019), Ramsey (1969), Baer & Leyer (2018)
	PBC2	Your company has the resources to build a blockchain base sustainable supply chain management system for SMEs in Malaysia	
Intention to use	BI1	SMEs will use blockchain very well in Malaysia	Amoako-Gyampah & Salam (2004), Lee (2009)
	BI2	It is predictable that SMEs will benefit from its applications in production and service processes	

### Data collection

An online survey method was applied to save time and get fast feedback from the respondents. Google Forms was used to get responses through email from the experts. The questionnaires were accessible online for a 16 week duration (mid-January 2023–mid-April 2023). In total, we obtained only 206 responses from experts working in the SMEs of Malaysia. The average completion period was approximately five minutes, close to the expected completion time based on the pilot testing. We have deleted 12 replies that were complete in less than one and a half minutes. Moreover, all responses were tested, and we uncovered that no answers to all items had the same grade. Due to missing data, further eight responses were withdrawn. Finally, for PLS-SEM, a total of 186 correct responses were considered. To determine the required sample size for testing our proposed model, we utilized the G* Power 3.1 software. We selected the setting for an F test with linear multiple regression, specifically the fixed model, where the R square increases. The parameters we used were as follows: an effect size (f square) of 0.15, an alpha error probability of 0.05, and a desired power of 0.95. Additionally, our model included a total of eight predictors. Based on these specifications, the calculated sample size required for testing our proposed model was determined to be 107. The study of Sideridis et al. (2014) has acclaimed that a small sample size is sufficient for PLS-SEM analysis. The sample size met the five observations per parameter norms. The demographic variables are presented in Table 3.

**Table 3:** Respondents profile (*N* = 186).

Profile		Frequency	Percentage
Gender	Male	99	53.2%
	Female	87	46.8%
Age	18–25 years	62	33.3%
	26–30 years	47	25.3%
	31–35years	39	21.0%
	36–40	29	15.6%
	41 above	9	4.8%
Job	Inventory managers	57	30.6%
	Operation managers	41	22.0%
	Finance managers	39	21.0%
	Research & development managers	37	19.9%
	IT officers	12	6.5%
Employees in SMEs	50–75	25	13.4%
	76–150	25	13.4%
	151–200	47	25.3%
	201–250	60	32.3%
	251–275	29	15.6%
Experience	≤2 years	29	15.6%
	>3 ≤ 5 years	26	14.0%
	>6 ≤ 10 years	46	24.7%
	>11 ≤ 15 years	53	28.5%
	>16 years	32	17.2%

### Method bias issues

We conducted a Harman’s single factor test (Chang, Witteloostuijn & Eden, 2010) to check the common method bias. Harman’s single factor test shows that 45% was the highest difference explained by a single variable. So, there is no multicollinearity problem in the data. Then, the variance inflation factor (VIF) test was used to determine the presence of excessively correlated factors before evaluating the model. The highest VIF score (3.705) was confirmed to be less than the standard value of 5 (Hair et al., 2019). The results show that this study does not have multicollinearity issues and that the measurement model is adequate. The VIF test results are presented in Table 4. Recursively from the structural model, endogeneity can be created. Consequently, we applied the Ramsey regression equation error test (Ramsey, 1969), and there was no endogeneity issue.

**Table 4:** VIF test.

CODE	Inner	Constructs	Outer								
ATT1	1.984		ATT	CPT	CPX	BI	PBC	PEOU	PU	RAD	SN
ATT2	2.197	Attitude				3.698					
ATT3	2.221	Compatibility						1.290	1.915		
ATT4	2.136	Complexity						1.635	1.636		
BI1	2.031	Intention to use									
BI2	2.031	Perceived behavioral control				2.588					
CPT1	1.699	Perceived ease of use	3.705						2.255		
CPT2	1.512	Perceived usefulness	3.705			3.012					
CPT3	1.858	Relative advantage						1.492	1.615		
CPT4	1.513	Subjective norms				2.747			2.382		
CPX1	1.732										
CPX2	2.081										
CPX3	1.415										
PBC1	1.616										
PBC2	1.616										
PEOU1	1.597										
PEOU2	1.959										
PEOU3	1.597										
PEOU4	1.547										
PU1	2.046										
PU2	2.059										
PU3	2.037										
PU4	2.321										
RAD1	1.506										
RAD2	1.634										
RAD3	1.477										
RAD4	1.488										
SN1	1.518										
SN2	2.017										
SN3	1.821										
SN4	1.440										

### Results

The proposed model was tested by a two-step process for finding a relationship among constructs. In the first phase, we tested the constructs’ reliability and validity, and in the second phase, the structural equation modeling was analyzed.

#### Reliability and validity

The study of Saunders, Lewis & Thornhill (2003) defines validity as the degree to which data gathering methods calculate specifically what they were designed to measure. In the present study, the subsequent reliability and validity tests were applied.

#### Convergent validity

A convergent validity test is practical when the hypothetical factor created for the analysis strongly correlates with the items utilized to measure it. There must be a significant discrepancy among the items in a construct. Nine constructs were investigated in this study using the rules for determining validity. The significant level of all factor loadings must be evaluated, and the value of all measurements must be more than 0.70.

- After validating the loadings, the composite reliability test must be performed on all of the factors; each construct’s value must be greater than 0.70 (Hair et al., 2016).
- Variance average: after confirming the reliability of each construct, it must be extracted and tested; the standard for each construct is 0.50 and above (Fornell & Larcker, 1981).
- The discriminant validity analysis examines the degree to which measurement factors in a proposed model are dissimilar from each other (Fornell & Larcker, 1981).

All of the tests for the study were completed using SmartPLS. The loading value for measurement item CPX4 was lower than expected (0.70), indicating a problem with internal consistency. As a result, the CPX4 component was removed from the research. Table 5 shows the factor loading values for each item.

**Table 5:** Outer loading.

	β	Mean	Standard deviation	T statistic	*P* values
ATT1 <- Attitude	0.827	0.829	0.023	35.669	0.000
ATT2 <- Attitude	0.847	0.849	0.022	39.309	0.000
ATT3 <- Attitude	0.849	0.847	0.022	38.826	0.000
ATT4 <- Attitude	0.846	0.845	0.021	40.330	0.000
BI1 <- Behavioral intention	0.925	0.925	0.011	85.098	0.000
BI2 <- Behavioral intention	0.926	0.926	0.010	89.784	0.000
CPT1 <- Compatibility	0.803	0.804	0.028	28.402	0.000
CPT2 <- Compatibility	0.739	0.734	0.039	18.910	0.000
CPT3 <- Compatibility	0.840	0.840	0.020	43.001	0.000
CPT4 <- Compatibility	0.778	0.778	0.035	22.147	0.000
CPX1 <- Complexity	0.846	0.845	0.032	26.036	0.000
CPX2 <- Complexity	0.867	0.864	0.027	31.991	0.000
CPX3 <- Complexity	0.758	0.750	0.047	15.979	0.000
CPX4 <- Complexity	0.499	0.482	0.103	4.860	0.000
PBC1 <- Perceived behavioral control	0.879	0.877	0.022	40.483	0.000
PBC2 <- Perceived behavioral control	0.918	0.917	0.011	80.349	0.000
PEOU1 <- Perceived ease of use	0.823	0.824	0.025	33.090	0.000
PEOU2 <- Perceived ease of use	0.822	0.822	0.024	34.405	0.000
PEOU3 <- Perceived ease of use	0.857	0.858	0.023	37.289	0.000
PEOU4 <- Perceived ease of use	0.827	0.827	0.023	35.197	0.000
PU1 <- Perceived usefulness	0.821	0.820	0.028	29.008	0.000
PU2 <- Perceived usefulness	0.849	0.850	0.022	39.369	0.000
PU3 <- Perceived usefulness	0.876	0.876	0.022	40.265	0.000
PU4 <- Perceived usefulness	0.841	0.842	0.020	41.788	0.000
RAD1 <- Relative advantage	0.748	0.743	0.051	14.534	0.000
RAD2 <- Relative advantage	0.781	0.782	0.036	21.501	0.000
RAD3 <- Relative advantage	0.750	0.745	0.044	16.912	0.000
RAD4 <- Relative advantage	0.806	0.806	0.031	25.577	0.000
SN1 <- Subjective norms	0.764	0.764	0.030	25.069	0.000
SN2 <- Subjective norms	0.835	0.835	0.028	30.112	0.000
SN3 <- Subjective norms	0.825	0.826	0.026	31.248	0.000
SN4 <- Subjective norms	0.739	0.737	0.036	20.610	0.000

**Figure 4:**
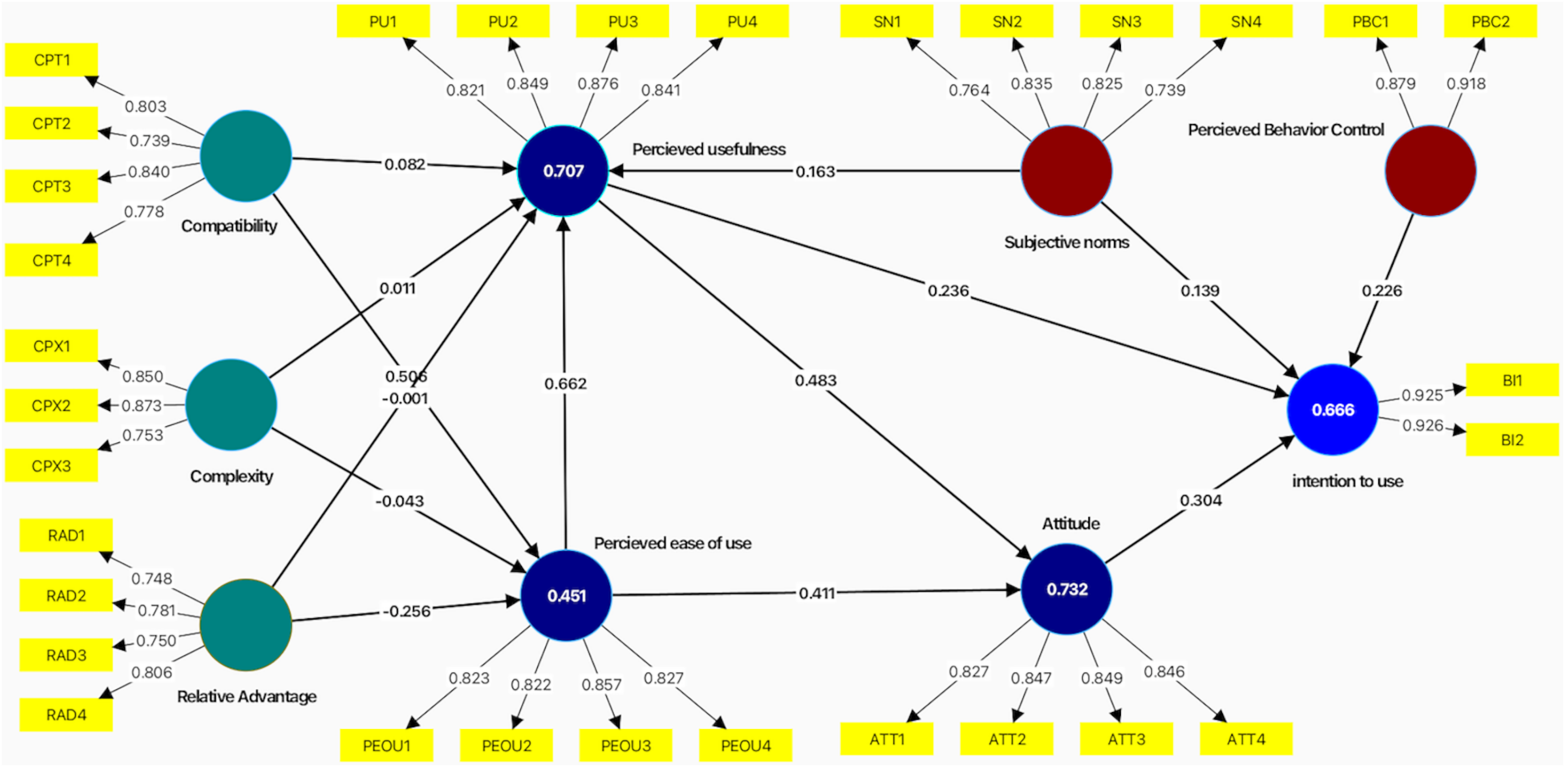
Measurement model.

After factor loadings, the composite reliability and average variance extracted were examined. Figure 4 depicts the measurement model. As shown in Table 6, all constructs were above the standard of 0.7, indicating a strong sign of measurement item consistency.

**Table 6:** Composite reliability and validity.

Code	Cronbach alpha	rho_A	Composite reliability	Average variance extracted
ATT	0.864	0.864	0.907	0.710
CPT	0.800	0.804	0.870	0.626
CPX	0.768	0.768	0.866	0.684
BI	0.832	0.832	0.923	0.856
PBC	0.764	0.781	0.893	0.808
PEOU	0.759	0.767	0.892	0.805
PU	0.850	0.851	0.909	0.769
RAD	0.775	0.777	0.855	0.597
SN	0.801	0.804	0.870	0.627

The discriminant validity of each latent variable was determined by testing the square root of average variance extracted, as indicated in Table 7. According to the data, average variance extracted square root is better than the association between the constructs, indicating that all items met the validity requirements and may be utilized to evaluate the structural equation model.

**Table 7:** Discriminant validity.

	ATT	CPT	CPX	BI	PBC	PEOU	PU	RAD	SN
ATT	**0.843**								
CPT	0.708	**0.791**							
CPX	−0.487	−0.456	**0.827**						
BI	0.771	0.616	−0.504	**0.925**					
PBC	0.741	0.647	−0.454	0.712	**0.899**				
PEOU	0.735	0.571	−0.367	0.680	0.616	**0.897**			
PU	0.793	0.576	−0.373	0.734	0.687	0.854	**0.877**		
RAD	−0.432	−0.364	0.562	−0.451	−0.369	−0.444	−0.427	**0.772**	
SN	0.751	0.632	−0.358	0.694	0.710	0.710	0.711	−0.373	**0.792**

**Note:**

The diagonals (bolded) are the square root of the AVE, while the off-diagonals are correlations.

#### Structural model

**Figure 5:**
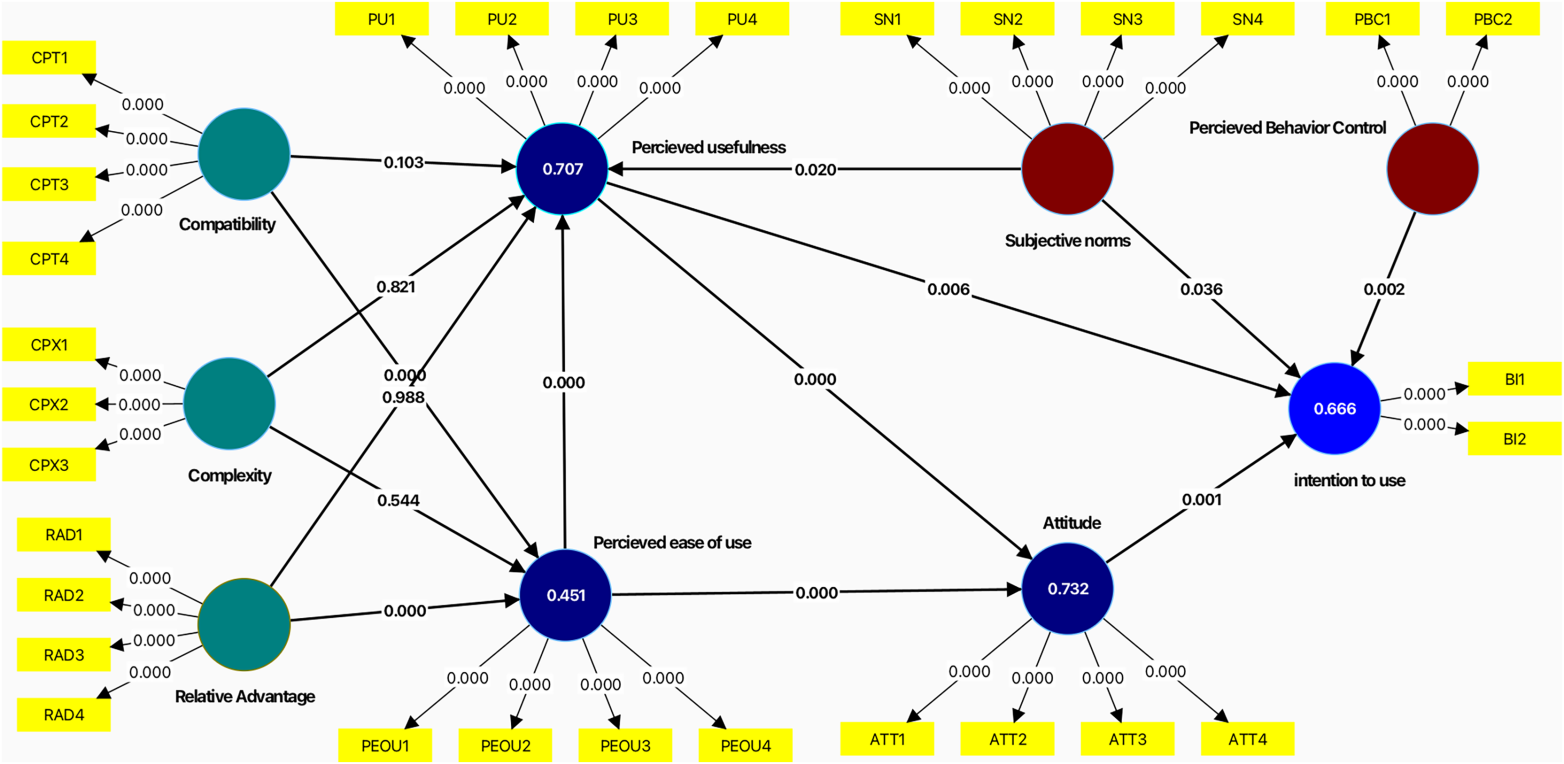
Structural model.

The significance assessment of the structural path was done using bootstrapping in the second phase of evaluating the structural equation modeling (SEM). The bootstrapping procedure involved testing several subsamples (5,000) from the original samples with replacements to check bootstrap faults. This led to the predicted T-values for measurement model significance testing. The bootstrapping procedure result estimates data normality, as shown in Fig. 5.

The findings confirm that the independent construct justified 66.6% of the variation on the intention to use. As a result, Table 8 shows the ultimate decision about hypothesis development.

**Table 8:** Hypothesis test result.

	Code	β	Mean	STDEV	T	*P*	Decision
H1	Perceived ease of use -> Perceived usefulness	0.675	0.672	0.056	12.156	0.000	Accepted
H2	Perceived ease of use -> Attitude	0.214	0.214	0.083	2.574	0.010	Accepted
H3	Perceived usefulness -> Attitude	0.610	0.612	0.076	7.990	0.000	Accepted
H4	Attitude -> Intention to use	0.328	0.332	0.084	3.918	0.000	Accepted
H5	Perceived usefulness -> Intention to use	0.235	0.229	0.074	3.176	0.002	Accepted
H6	Compatibility -> Perceived usefulness	0.060	0.061	0.058	1.041	0.298	Rejected
H7	Compatibility -> Perceived ease of use	0.473	0.478	0.078	6.069	0.000	Accepted
H8	Complexity -> Perceived usefulness	−0.017	−0.016	0.050	0.341	0.733	Rejected
H9	Complexity -> Perceive ease of use	0.001	0.002	0.085	0.015	0.988	Rejected
H10	Relative advantage -> Perceived usefulness	−0.030	−0.030	0.048	0.633	0.527	Rejected
H11	Relative advantage-> Perceived ease of use	−0.272	−0.276	0.081	3.381	0.001	Accepted
H12	Subjective norms -> Perceived usefulness	0.177	0.180	0.067	2.653	0.008	Accepted
H13	Subject norms -> Intention to use	0.124	0.127	0.069	1.797	0.073	Rejected
H14	Perceived behavioral control -> Intention to use	0.220	0.219	0.071	3.098	0.002	Accepted

Furthermore, we applied the partial least square (PLS) predict, a holdout sample-based technique that creates case level estimates on an item utilizing the PLS predict with a 10-fold process to test for predictive power. According to Table 9, the PLS model’s majority of errors were lower than the linear model’s (LM), representing that our proposed model has modest predictive power.

**Table 9:** PLS-predict.

	PLS		LM		PLS – LM		
	RMSE	MAE	RMSE	MAE	RMSE	MAE	Q^2^_predict
BI1	0.920	0.783	0.933	0.765	0.013	−0.018	0.489
B12	0.880	0.703	0.885	0.705	0.005	0.002	0.487

#### Structural model assessment

Based on the results presented in Table 8, the following structural model assessment can be made.

H1: Perceived ease of use has a positive relationship with perceived usefulness (β = 0.675, T = 12.156, *P* = 0.000). So, H1 is supported.

H2: Perceived ease of use has a significant relationship with attitude (β = 0.214, T = 2.574, *P* = 0.010). Thus, H2 is supported.

H3: Perceived usefulness has a significant relationship with attitude (β = 0.610, T = 7.990, *P* = 0.000). Hence, H3 is supported.

H4: Attitude has a significant relationship with intention to use (β = 0.328, T = 3.918, *P* = 0.000). Therefore, H4 is supported.

H5: Perceived usefulness has a significant relationship with intention to use (β = 0.235, T = 3.176, *P* = 0.002). Hence, H5 is supported.

H6: Compatibility does not have a significant relationship with perceived usefulness (β = 0.060, T = 1.041, *P* = 0.298). Thus, H6 is not supported.

H7: Compatibility has a significant relationship with perceived ease of use (β = 0.473, T = 6.069, *P* = 0.000). Therefore, H7 is supported.

H8: Complexity does not have a significant relationship with perceived usefulness (β = −0.017, T = 0.341, *P* = 0.733). Thus, H8 is not supported.

H9: Complexity does not have a significant relationship with perceived ease of use (β = 0.001, T = 0.015, *P* = 0.988). Hence, H9 is not supported.

H10: Relative advantage does not have a significant relationship with perceived usefulness (β = −0.030, T = 0.633, *P* = 0.527). So, H10 is not supported.

H11: Relative advantage has a significant relationship with perceived ease of use (β = −0.272, T = 3.381, *P* = 0.001). Therefore, H11 is supported.

H12: Subjective norms have a significant relationship with perceived usefulness (β = 0.177, T = 2.653, *P* = 0.008). Hence, H12 is supported.

H13: Subjective norms do not have a significant relationship with intention to use (β = 0.124, T = 1.797, *P* = 0.073). Thus, H13 is not supported.

H14: Perceived behavioral control has a significant relationship with intention to use (β = 0.220, T = 3.098, *P* = 0.002). Therefore, H14 is supported.

## Discussion

In this research, the focus was on understanding the factors that influence the adoption of blockchain technology. To achieve this, a sophisticated statistical technique called partial least square structural equation modeling (PLS-SEM) was utilized. The study integrated three well-established adoption models and formulated hypotheses based on them. Data was collected through an online survey from 186 supply chain experts working in SMEs in Malaysia. The PLS-SEM results showed that integrating three adoption theories is essential for the SME sector. The TAM constructs achieved the highest rank, indicating that it is the most significant factor in implementing blockchain in SMEs post-COVID-19. Many experts believe that adopting blockchain in SMEs can increase cost competence, improve stock replacement, consolidate marketing policy, and increase product safety. The study findings confirm that perceived ease of use positively is positively associated with perceived usefulness and is supported by the prior studies of Lisha et al. (2017) and Yoon (2016). Similarly, perceived ease of use is positively associated with attitude and supported by the studies of Gao & Bai (2014) and Baer & Leyer (2018). Moreover, the findings show that perceived usefulness is significantly associated with attitude, as supported by the prior studies of Amoako-Gyampah & Salam (2004) and Lee (2009). The attitude shows a positive and substantial relationship with intention to use blockchain and is supported by the previous study of Kamble, Gunasekaran & Arha (2019). The next perceived usefulness is also significantly associated with the intention to use blockchain and is supported by the other study of Yoon (2016). Based on the findings, it can be understood that technology acceptance model constructs play a critical role in adopting innovative technology. Blockchain acceptance in SMEs can aid supply chain integration, reliable IT framework, product safety, quick data sharing, cooperation development and process integration. However, the diffusion of innovation construct compatibility shows an insignificant relationship with perceived usefulness and has been contradicted by the previous study of Lee, Hsieh & Hsu (2011), while being supported by the research of Al-Rahmi et al. (2019). In addition, compatibility is significantly associated with the perceived ease of use and is supported by the prior research of Lee, Hsieh & Hsu (2011). The results further specify that complexity shows an insignificant relationship with perceived usefulness and perceived ease of use and is contradicted by the previous study of Alalwan et al. (2019), while being supported by the other research of Lee, Hsieh & Hsu (2011). Moreover, relative advantage shows an insignificant relationship with the perceived usefulness, and is contradicted by the earlier studies of Alalwan et al. (2019) and Lee, Hsieh & Hsu (2011). Conversely, relative advantage shows a significant relationship with the perceived ease of use and is supported by the other studies of Al-Rahmi et al. (2019) and Lee, Hsieh & Hsu (2011). The study findings further indicate that the theory of planned behavior construct, subjective norms, is positively associated with the perceived usefulness, and supported by the other study of Kamble, Gunasekaran & Arha (2019). However, subjective norms show an insignificant relationship with the intention to use and supported by the other studies of Kamble, Gunasekaran & Arha (2019) and Sentosa & Mat (2012). Consequently, the study findings confirm that perceived control is significantly associated with the intention to use, which is supported by the other studies of Baker, Al-Gahtani & Hubona (2007) and Ajzen (1991).

### Theoretical implications

Our study represents a significant contribution to the field of information systems adoption and sustainable SCM by proposing a model to investigate the acceptance of blockchain in post-COVID-19 sustainable SCM systems of SMEs in Malaysia. Unlike the predominantly conceptual nature of existing research on blockchain, our study stands out as an empirical analysis that integrates traditional adoption theories, including the technology acceptance model, diffusion of innovation theory, and theory of planned behavior. This integration allows us to gain a comprehensive understanding of the factors influencing the adoption of blockchain technology. Importantly, there is a lack of empirical research focusing on developing economies like Malaysia in this area. By utilizing constructs derived from these adoption theories, such as perceived ease of use, perceived usefulness, attitude, complexity, compatibility, relative advantage, subjective norms, and perceived behavior control, our study fills a gap in the literature and provides valuable insights into the acceptance of disruptive technologies in SMEs’ sustainable SCM systems. The findings of our proposed model contribute to the body of knowledge on innovation adoption and offer practical implications for academics and experts seeking to understand and implement blockchain technology in the post-COVID-19 sustainable SCM of SMEs.

### Practical implications

The findings of our proposed model have significant practical implications, as they demonstrate a strong explanatory power, accounting for 66.6 percent of the variation in the intention to use BCT in SMEs post-COVID-19. These implications provide valuable insights and realistic recommendations for organizations seeking to address critical sustainable SCM issues through the adoption of blockchain technology. For SMEs in emerging economies considering the adoption of BCT, it is crucial to grasp the fundamental constructs of behavioral intention identified in our study. Additionally, BCT developers should not only focus on understanding the technology itself, but also develop BCT applications specifically designed for sustainable SCM systems. These applications can enable retailers to track stock in real-time, enhance replenishment efficiency, reduce product misplacement, and lower labor costs. Moreover, BCT implementation can assist administrations in organizing anti-counterfeit measures in the post-COVID-19 era. Based on the results of our study, the conceptual model demonstrates its descriptive power and provides a holistic approach for future research on information system implementation. For experts implementing BCT, our study offers valuable insights into developing innovative technological solutions. BCT implementation can promote transparency in the manufacturing and service industries, increase cost-effectiveness, centralize marketing strategies, enable organizations to swiftly obtain customer information, and contribute to gaining a competitive edge in the post-COVID-19 business landscape.

### Conclusion and future work

This study explores the factors influencing the adoption of BCT in SMEs in the post-COVID-19 era. The study utilizes the unified framework of three traditional theories: technology acceptance model (TAM), diffusion of innovation (DOI), and theory of planned behavior (TPB). The study examines the relationship of perceived usefulness, perceived ease of use, attitude, relative advantage, compatibility, subjective norms, and perceived behavioral control on the intention to adopt BCT in SMEs for post-COVID-19. In response to Research Question 01, the results indicate that perceived usefulness, perceived ease of use, and attitude is significantly associated with the intention to accept BCT in SMEs. Additionally, relative advantage and compatibility have a significant relationship with perceived ease of use. Subjective norms is significantly associated with perceived usefulness, while perceived behavioral control is substantially associated with the intention to use BCT in SMEs in Malaysia. However, the complexity does not show a significant relationship with either perceived usefulness or perceived ease of use. In response to Research Question 02, the study also investigates the importance of TAM constructs (perceived usefulness, ease of use, and attitude) during BCT adoption in SMEs for sustainable SCM systems. The findings reveal that TAM construct perceived ease of use is most crucial in this context. However, compatibility, relative advantage, and subjective norms show no significant relationship with perceived usefulness. Moreover, subjective norms have a negligible relationship with intention to use blockchain technology.

While this study provides valuable insights, there are some limitations that open avenues for further research. Firstly, the study is based on the opinions of Malaysian SME practitioners, and perspectives from practitioners in other countries may differ. Secondly, the sample size is limited to Malaysia, so caution must be exercised when generalizing the findings to other developed countries like the US and UK in the post-COVID-19 context. Thirdly, the practical implementation of blockchain technology in developing economies like Malaysia is still limited, and further research is needed to understand post-adoption behavior. Fourthly, blockchain is not a standalone technology, and future research can explore its integration with AI and IoT to address security, privacy, and data integration concerns. Fifthly, local regulations play a significant role in technology adoption, and considering them will enhance the validity of future research in the post-COVID-19 era. Finally, other factors such as cost, traceability, self-control, perceived enjoyment, information quality, and system quality should be included in future research to gain a comprehensive understanding of blockchain adoption in sustainable SCM for post-COVID-19 SMEs. Overall, this analysis provides valuable insights into the critical success factors for BCT adoption and its impact on sustainable supply chain management in SMEs. Further research in these areas will contribute to a better understanding of the topic and its implications.

## Supplemental Information

10.7717/peerj-cs.2466/supp-1 Supplemental Information 1 Data.

## Additional Information and Declarations

### Data Availability

The following information was supplied regarding data availability:

The data are available in the Supplemental Files.
